# Comparison of four scoring systems for risk stratification of upper gastrointestinal bleeding

**DOI:** 10.12669/pjms.343.14956

**Published:** 2018

**Authors:** Hakan Tuncer, Turker Yardan, Hizir Ufuk Akdemir, Talat Ayyildiz

**Affiliations:** 1 Dr. HakanTuncer, MD. Department of Emergency Medicine, Bagcilar Education and Research Hospital, Istanbul, Turkey; 2 Dr. TurkerYardan, MD. Department of Emergency Medicine, Associate Professor, Faculty of Medicine, Ondokuz Mayis University, Samsun, Turkey.; 3 Hizir Ufuk Akdemir, MD. Department of Emergency Medicine, Associate Professor, Faculty of Medicine, Ondokuz Mayis University, Samsun, Turkey; 4 Talat Ayyildiz, MD. Department of Gastroenterology, Associate Professor, Faculty of Medicine, Ondokuz Mayis University, Samsun, Turkey

**Keywords:** Gastrointestinal bleeding, High-Risk, Mortality, Rebleeding, Scoring systems

## Abstract

**Objective:**

This study aimed to compare the performances of the Glasgow–Blatchford Bleeding Score (GBS), pre-endoscopic Rockall score (PRS), complete Rockall score (CRS), and Cedars–Sinai Medical Center Predictive Index (CSMCPI) in predicting clinical outcomes in patients with upper gastrointestinal bleeding (UGIB).

**Methods:**

Patients who were admitted to the emergency department because of UGIB and underwent endoscopy within the first 24 hour were included in this study. The GBS, PRS, CRS, and CSMCPI were propectively calculated. The performances of these scores were assessed using a receiver operating characteristic curve.

**Results:**

A total of 153 patients were included in this study. For the prediction of high-risk patients, area under the curve (AUC) was obtained for GBS (0.912), PRS (0.968), CRS (0.991), and CSMCPI (0.918). For the prediction of rebleeding, AUC was obtained for GBS (0.656), PRS (0.625), CRS (0.701), and CSMCPI (0.612). For the prediction of 30-day mortality, AUC was obtained for GBS (0.658), PRS (0.757), CRS (0.823), and CSMCPI (0.745).

**Conclusion:**

These results suggest that effectiveness of CRS is higher than that of other scores in predicting high-risk patients, rebleeding and 30-day mortality in patients with UGIB.

## Introduction

Acute upper gastrointestinal bleeding (UGIB) is managed on an inpatient basis, with emergency departments (ED) usually diagnosing the condition and initiating treatment.^1^ The clinical severities of UGIB) are various, ranging from insignificant bleeding to fatal outcomes.^2^ Bleeding generally stops spontaneously in over 80% of cases with no need for intervention. Therefore, low-risk patients may be more efficiently managed in the community and do not require hospital admission.^1^

Accurate identification of high-risk patients can help physicians decide on hospital admission or discharge, the level of assistance (early endoscopy or not), and the type of treatment (medical, endoscopic, or surgical intervention)in UGIB.^2^ In recent years, several practice guidelines and risk scores that combine clinical and endoscopic parameters have been developed to assist physicians in the early stages of decision making.^3^ The most widely quoted are the Glasgow-Blatchford Bleeding Score (GBS)^4^,^5^ and the pre-endoscopic Rock all score (PRS)^6^,^7^, both of which consider only pre-endoscopy criteria, and the complete Rockall score (CRS)^8^,^9^ and the Cedars–Sinai Medical Center Predictive Index (CSMCPI),^10^-^12^ both of which have additional endoscopic criteria. However, whether any of these tools are sufficiently predictive to serve as a decision guide for emergency physicians remains unclear.^13^

This study aimed to compare the performances of GBS, PRS, CRS, and CSMCPI for the prediction of high-risk patients, rebleeding, and 30-day mortality in patients with acute UGIB in the ED.

## Methods

We prospectively studied all adult patients presenting with UGIB who were admitted to the ED of Ondokuz Mayis University, Faculty of Medicine, in Samsun, Turkey, between January 2014 and December 2014. The study protocol was approved by the local ethics committee (serial number 2013/452), and informed consent was obtained from all enrolled patients.

All nontrauma adult (i.e., >18 years old) patients with UGIB admitted to the ED were evaluated. The diagnosis of UGIB was based on patients’ presentations, including coffee ground vomit, hematemesis, melena, and blood in nasogastric aspirate.^2^ These patients were considered eligible for the study at the time of UGIB diagnosis. Patients with UGIB who received initial endoscopy within 24 hour of presentation were enrolled in the study. The data were prospectively collected by a research student using standardized data collection forms. *High-risk* patients were defined as those requiring blood transfusion, therapeutic endoscopy to control bleeding, or surgical intervention to control bleeding. *Rebleeding* patients were defined as those with any of the following:

1. Repeated endoscopy within three days
2. Continuous blood transfusion for more than three days
3. Surgical intervention to control bleeding within three days.^2^

All clinical management decisions were conducted by emergency physicians, and gastroenterology consultation was obtained for all patients. The standard management for all patients with non-variceal UGIB in our ED is the administration of an intravenous proton pump inhibitor before endoscopy. Intravenous somatostatin or octreotide was started in all patients with suspected variceal bleeding. The decision and timing of the endoscopy were at the discretion of the gastroenterologists. Blood transfusion was indicated for UGIB patients with hemoglobin of less than 10 g/dL or with signs of hemodynamic instability despite fluid resuscitation.^2^ Surgical consultation was obtained if appropriate medical, including endoscopic, therapies failed. Patients were admitted to the emergency intensive care unit (ICU) or ward based on their response to initial management. Patients without evidence of active bleeding and in stable condition were discharged if there was no more UGIB.

### Evaluating the scoring systems

The GBS^4^,^5^, PRS^6^,^7^, CRS^8^,^9^, and CSMCPI^10^-^12^ scores were calculated for all patients according to the criteria stated in the original articles. According to the original articles, the cut-off values used for the prediction of high-risk patients were GBS>0, PRS>0, CRS>2, and CSMCPI ≥5. The performances of these cut-off values for the prediction of high-risk patients, rebleeding, and 30-day mortality were compared.

### Statistical analysis

Statistical analyses were conducted using SPSS 20.0 for Windows (SPSS Inc., Chicago, Illinois, USA). Data were analyzed with the Kolmogorov–Smirnov test for normality. Values were reported as median (minimum–maximum) for data that were not distributed normally. The Mann–Whitney U test and χ^2^ or the Fisher exact test were used to compare the statistically significant differences between high-risk and non-high-risk patients. A value of p<0.05 was accepted as statistically significant. The receiver operating characteristic (ROC) curves were calculated to identify the GBS>0, PRS>0, CRS>2, and CSMCPI ≥5 cut-off values for predicting high-risk patients, rebleeding, and 30-day mortality. Sensitivity, specificity, positive predictive value, and negative predictive value with their 95% confidence intervals were calculated.

## Results

During the study period, 203 patients presented with UGIB in the ED. Among these patients, 30 who underwent endoscopy after 24 hour of presentation were excluded from the study, Moreover, three patients who underwent emergency surgery and 17 who did not accept endoscopy were excluded from the study.

A total of 153 patients who underwent endoscopy for UGIB within 24 hour of presentation in the ED were enrolled in this study. The median time elapsed from the ED triage to endoscopy was 13 h (1–24). Peptic ulcer was found in 72 (47.1%) patients and esophageal varices or gastric varices were found in 32 (20.9%) patients. The baseline characteristics of patients with UGIB and the comparison between high-risk patients and non–high-risk patients are shown in Table-I.

**Table-I:** Baseline characteristics of patients with upper gastrointestinal bleeding and the comparison between high-risk and non-high-risk patients.

	All patients (n=153)	High risk patients (n=134)	Non–high risk patients (n=19)	P-value
Age (years)	64 (20-92)	65.5 (20-92)	48 (20-81)	<0.05
Male	103 (67.3)	88 (65.7)	15 (78.9)	>0.05
Symptoms				
Melena	92 (60.1)	105 (78.4)	13 (64.8)	>0.05
Hematemesis	60 (39.2)	54 (40.3)	6 (31.6)	>0.05
Weakness	50 (32.7)	48 (35.8)	2 (10.5)	>0.05
Syncope	8 (5.2)	8 (6)	0 (0)	>0.05
Epigastric pain	6 (3.9)	6 (4.5)	0 (0)	>0.05
Dyspnea	9 (5.9)	9 (6.7)	0 (0)	>0.05
Previous medication				
NSAID use	25 (16.3)	20 (14.9)	3 (15.8)	>0.05
Clopidogrel use	2 (1.3)	2 (1.5)	0 (0)	>0.05
Warfarin use	15 (9.8)	14 (10.4)	1 (5.3)	>0.05
Aspirin use	24 (15.7)	23 (17.2)	1 (5.3)	>0.05
Comorbidity				
Heart failure	38 (24.8)	38 (28.4)	0 (0)	<0.05
Malignancy	10 (6.5)	10 (7.5)	0 (0)	>0.05
Cirrhosis	30 (19.6)	29 (21.6)	1 (5.3)	>0.05
UGIB history	46 (30)	43 (32.1)	3 (15.8)	>0.05
***Initial laboratory tests***				
Hemoglobin (g/dl)	8.3 (4.1-16)	7.9 (4.1-13)	11 (8.3-16)	<0.05
Hematocrit	25 (13-46)	24 (13-40)	34 (25-46)	<0.05
Platelet count (1000/µl)	218 (12-982)	205 (12-594)	273 (53-982)	<0.05
INR	1.1 (0.9-9.8)	1.2 (0.9-9.8)	1.1 (1-1.3)	<0.05
GBS	13 (4-19)	13 (5-19)	8 (4-13)	<0.05
PRS	3 (0-7)	4 (0-7)	0 (0-2)	<0.05
CRS	5 (0-10)	5 (1-10)	1 (0-2)	<0.05
CSMCPI	4 (0-8)	4 (0-8)	1 (0-4)	<0.05

Data are presented as n (%) or median (min–max). NSAID: Nonsteroidal Anti-Inflammatory Drug;

UGIB: Upper Gastrointestinal Bleeding; INR: International Normalized Ratio.

A total of 122 (79.7%) patients needed blood transfusion during ED or hospital stay, 76 (49.7%) patients needed endoscopic treatment to control bleeding, and 2 (1.3%) patients needed surgical treatment to control bleeding.

A total of 134 patients (87.6%) comprised the high-risk group, 28 (18.3%) patients rebled, and 9 (5.9%) patients died after a 30-day follow-up. About 104 (68%) patients were treated in the ward, 37 (24.2%) patients were treated in the ICU, and 12 (7.8%) patients were treated as outpatients. The median duration of hospitalization was 4 (1–35) days.

In the ROC analysis to predict high-risk patients, the area under the curve (AUC) was obtained for GBS (0.912; 95% CI, 0.855–0.970; p<0.001), PRS (0.968; 95% CI, 0.939–0.996; p<0.001), CRS (0.991; 95% CI, 0.979–1.000; p<0.001), and CSMCPI (0.918; 95% CI, 0.850–0.986; p<0.001).

For the prediction of rebleeding, the AUC was obtained for GBS (0.656; 95% CI, 0.557–0.756; p=0.01), PRS (0.625; 95% CI, 0.514–0.735; p=0.039), CRS (0.701; 95% CI, 0.607–0.795; p=0.001), and CSMCPI (0.612; 95% CI, 0.508–0.717; p=0.064).

For the prediction of 30-day mortality, the AUC was obtained for GBS (0.658; 95% CI, 0.504–0.812; p=0.112), PRS (0.757; 95% CI, 0.597–0.918; p=0.01), CRS (0.823; 95% CI, 0.708–0.937; p=0.001), and CSMCPI (0.745; 95% CI, 0.615–0.874; p=0.014). The sensitivity and specificity of GBS>0, PRS>0, CRS>2, and CSMCPI ≥ 5 in detecting the high-risk group, rebleeding, and 30-day mortality are presented in Tables II, III, and IV, respectively.

**Table-II:** Sensitivity and specificity of GBS>0, PRS>0, CRS>2, and CSMCPI≥5 in detecting high-risk patients.

	Sensitivity (95% CI)	Specificity (95% CI)	Positive predictive value (95% CI)	Negative predictive value (95% CI)
GBS>0	100 (97.3–100)	0 (0–17.7)	87.6 (81.3–92.4)	-
PRS >0	98.5 (97.4–99.8)	68.4 (43.5–87.4)	95.7 (90.8–98.4)	86.7 (59.5–98.3)
CRS >2	94.8 (89.5–97.9)	100 (82.4–100)	100 (94.1–100)	73.1 (52.2–84.4)
CSMCPI ≥5	47.8 (39.1–56.6)	100 (82.4–100)	100 (94.4–100)	21.4 (13.4–31.3)

**Table-III:** Sensitivity and specificity of GBS>0, PRS>0, CRS>2, and CSMCPI≥5 in detecting rebleeding.

	Sensitivity (95% CI)	Specificity (95% CI)	Positive predictive value (95% CI)	Negative predictive value (95% CI)
GBS>0	100 (87.7–100)	0 (0–2.9)	18.3 (12.5–25.4)	-
PRS >0	100 (87.7–100)	12 (6.9–19)	20.3 (13.9–28)	100 (78.2–100)
CRS >2	96.4 (81.7–99.9)	20 (13.4–28.1)	21.3 (14.5–29.4)	96.2 (80.4–99.9)
CSMCPI ≥5	53.6 (33.9–72.5)	60.8 (51.7–69.4)	23.4 (13.8–35.7)	85.4 (76.3–92)

**Table-IV:** Sensitivity and specificity of GBS>0, PRS>0, CRS>2, and CSMCPI≥5 in detecting 30-day mortality.

	Sensitivity (95% CI)	Specificity (95% CI)	Positive predictive value (95% CI)	Negative predictive value (95% CI)
GBS>0	100 (66.4–100)	0 (0–2.5)	5.9 (2.7–10.9)	-
PRS >0	100 (66.4–100)	10.4 (6–16.6)	6.5 (3–12)	100 (78.2–100)
CRS >2	100 (66.4–100)	18.1 (12.2–25.3)	7.1 (3.3–13)	100 (86.8–100)
CSMCPI ≥5	77.8 (40–97.2)	60.4 (51.9–68.5)	10.9 (4.5–21.2)	97.8 (92.1–99.7)

## Discussion

The early identification of patients at high risk for mortality and rebleeding can help improve both efficiency of care and potentially outcomes for patients. Moreover, the early identification of patients who would require endoscopic, radiological, or surgical intervention could allow for better allocation of resources.^14^ To the best of our knowledge, this study is the first comparing the performance of the four scoring systems as early risk-assessment tools in ED patients with UGIB.

In the present study, the scoring systems showed high AUC values for the detection of high-risk patients. The AUC of CRS (0.991) was higher than that of GBS (0.912), PRS (0.968), and CSMCPI (0.918). The sensitivity of CSMCPI and the specificity of GBS were low in the prediction of high-risk patients. In our study, the minimum GBS value was 4. Thus, no negative predictive value was found for GBS>0. This finding may be related to the excluded patients who were generally stable and received endoscopy 24 hour after admission. If we consider this bias, the number of patients who scored 0 on the GBS will increase. Similarly, previous studies have reported that the number of patients who scored 0 is relatively small (0.8%–4.6%).^15^,^16^ Potential cut-off GBS scores may be found, and the higher cut-off GBS value (≤3) than zero may be more useful in the risk stratification of hospitalized patients with UGIB.^17^

Our study shows that CRS is superior to GBS, PRS, and CSMCPI in predicting high-risk patients. In contrast to our results, Wang CH et al.^2^ reported that the AUC of GBS was higher than that of CRS and PRS in the prediction of high risk patients. However, they considered the CRS to be more accurate because all of their enrolled patients received endoscopy. Similarly, GBS was reported to be more useful than PRS and CRS in predicting the need for blood transfusion and endoscopic or surgical intervention.^14^ In a recently published study, GBS was reported to be equivalent to CRS and superior to PRS in predicting the need for hospital-based intervention.^15^

In the present study, for the prediction of rebleeding, the AUC of CRS (0.701) was higher than that of GBS (0.656), PRS (0.625), and CSMCPI (0.612). CSMCPI was not effective in the prediction of rebleeding (p=0.064). Laursen SB et al.^18^ reported that CSMCPI did not accurately predict patients’ 30-day mortality or rebleeding. Similar to our results, Yang HM et al.^15^ reported CRS to be superior to both GBS and PRS for the prediction of rebleeding. However, similar to our results, the specificity of GBS, CRS, and PRS was found to be low in previous studies.^2^,^15^ CRS, GBS, and PRS had high sensitivity in predicting rebleeding (Table-III). However, routine use in predicting rebleeding should be considered because of the low specificity of this systems.^2^

For the prediction of 30-day mortality, the AUC of CRS (0.823) was higher than that of PRS (0.757) and CSMCPI (0.745). GBS was not effective in predicting mortality (p=0.112) (Table-IV). CRS was reported to have an acceptable performance in predicting mortality.^19^ Similar to our results, Cassana A et al.^20^ reported that GBS has no diagnostic validity in predicting mortality in UGIB. Wang CH et al.^2^ found that GBS, CRS, and PRS have no good performance in predicting 30-day mortality in UGIB. The present study suggests that CRS is superior to the other scoring systems in predicting 30-day mortality. However, the application of CRS, PRS, and CSMCPI in predicting mortality should be made with caution because of their low specificity.

### Limitations

This study has several limitations. First, similar to previous studies^2^,^21^, the present study included patients with UGIB and calculated all four scores only in patients with confirmed UGIB diagnosis through endoscopy. Patients who did not receive endoscopy and patients with UGIB who received endoscopy 24 hour after admission were excluded. The inclusion of all individuals with UGIB symptoms might have provided more reliable results. Second, this study was conducted in a single center. However, many patients with UGIB from other hospitals were sent to our ED, in which 24-hour emergent endoscopy and a surgeon were available. Lastly, while assessing the scores, we did not consider the potential effect of bleeding sources on the scores.

## CONCLUSIONS

The effectiveness of CRS was higher than that of other scores in predicting high-risk patients, rebleeding and 30-day mortality in patients with UGIB. CSMCPI was not effective in the prediction of rebleeding, and GBS was not effective in the prediction of 30 day- mortality. Our data suggest that CRS performs well as a scoring system and should be the scoring system of choice in the assessment of patients with UGIB in the ED. In the application of GBS, PRS,CRS, and CSMCPI for risk stratification in UGIB, emergency physicians should consider their predictive performance in clinical practice.
